# Surgical results and quality of life after single-stage posterior transpedicular approach for circumferential epidural decompression in patients with thoracolumbar spine metastasis

**DOI:** 10.7150/jca.85939

**Published:** 2023-07-09

**Authors:** Jianfang Niu, Zhiqing Zhao, Jichuan Wang, Taiqiang Yan, Wei Guo, Rongli Yang, Xiaodong Tang

**Affiliations:** Musculoskeletal Tumor Centre, Peking University People's Hospital, 11# Xizhimen South Road, Xicheng District, Beijing, China, 100044.

**Keywords:** spinal metastases, surgery, quality of life

## Abstract

**Objective:** The primary aim of this study was to evaluate the effect of palliative surgery using posterior transpedicular approach (PTA) with posterior instrumentation on pain response and quality of life (QoL) in patients with metastatic thoracic and lumbar tumors.

**Methods:** From 2018 to 2019, 39 patients with metastatic thoracic and/or lumbar tumors were prospectively enrolled to measure the reduction in pain and the changes in QoL after surgical decompression with posterior instrumentation via PTA. The patient group was composed of 27 men and 12 women with a mean age of 60 years (range, 28 to 92 years). Pain response was measured using the visual analog scale (VAS) and neurologic status was evaluated using Frankel grades. QoL was assessed with use of the EORCT QLQ-BM22 questionnaire before surgery (baseline) and at 1-, 3-, 6-, and 12-month after surgery. The survival times of all the patients were also collected.

**Results:** All patients showed either an improvement or a similar pain level after surgery, which the VAS score decreased from 7.10 ± 2.22 preoperatively to 3.10 ± 2.15 one month postoperatively (*P*<0.05). 19 patients (48.7%, 19/39) showed neurological function improvement postoperatively. Among the 19 patients, 7 cases improved from Frankel grade C to D, 5 cases from grade C to E, and 7 cases from grade D to E. Another 20 patients still have the same Frankel grade postoperatively, however, most of them improved clinically. The QoL improvement of the patients was also evident after treatment. Paired-samples T-test examination of the postoperative scores showed a significant improvement in terms of pain location, pain severity and performance status (*P*<0.01). Compared with the preoperative score, the 1-month postoperative score of functional interference was significantly improved (63.6 vs. 34.5, *P*<0.01). There were no significant changes in social or psychological functioning. Three patients experienced cerebrospinal fluid leakage postoperatively, and they were all successfully managed by lying flat without a pillow. One patient experienced rod breakage, at 10 months after surgery. All the patients were alive at 3 months; however, 7 patients died within 3 to 6 months, and another 9 patients died from the disease within 6 to 12 months.

**Conclusions:** The present feasibility study found that the application of the PTA for decompression and fusion in patients with spinal metastases is beneficial for achieving prompt and sustained pain relief, reducing neurologic deficits and improving functional outcomes, health utilities, and HRQoL.

## Introduction

The spine is the most common boney location for cancer metastases 1, 2. Approximately, spinal metastases presents in 30% to 40% of patients with malignant tumor^3^, ^4^, and it is expected that a group of these patients have symptomatic metastatic lesions of the spinal column. Epidural compression caused by mechanical instability will happen in 5% to 20% of patients, which leading to clinical symptoms, such as neurological dysfunction and intractable pain^3^. These symptoms can gradually result in severe deterioration in the patients' quality of life (QoL) 2, 5, 6. Therapeutic options for metastatic lesions in spine include radiotherapy, external irradiation, minimally surgery, or open surgery. In certain circumstances, for those with intractable pain, worsening neurological function, or instabilities of spine, radioiodine, and external irradiation may not produce quick outcomes. Therefore, surgical treatment should be taken into consideration if the patient has a good general state. Minimally invasive procedures including vertebroplasty and kyphoplasty can be performed to achieve pain relief and restore some degree of stability, however, they do not allow for the tumor resection and resolve the epidural compression.

Overall, those with metastatic spinal tumors are usually older, and have diminished pulmonary function and general state. Consequently, the anterior approach is not recommended for these patients^7^. To decrease the morbidity encountered in the combined anterior-posterior surgical procedure, circumferential decompression with a single posterolateral transpedicular approach (PTA) can be performed^8^-^10^. Surgery for spinal metastases is a palliative option, and the goals of treatment are to improve and maintain patients' activity of daily living and QoL until the end-state. Patient reported outcome (PRO) measures are important for analyzing the effect of disease and therapy on patient symptoms 11. Therefore, this prospective study aim to use validated PRO measurements and pain outcomes to assess the efficacy of single-stage PTA for circumferential epidural decompression and posterior instrumentation.

## Materials and Methods

This prospective clinical study was approved by the Ethical Review Committee of Peking University People's Hospital (No. 2020PHB149-01). Patients were prospectively enrolled from January 2018 to December 2019. The inclusion criteria were as follows: (i) patients who were over 18 years with a sober consciousness and has the ability to understand and fill in the questionnaires; (ii) patients with thoracolumbar vertebral metastases suffered severe pain, pathologic fractures and metastatic epidural spinal cord compression (MESCC). Patients with radio-resistant or previously radiated spinal lesions were also included in our study. Patients who could not tolerate operation or who had a predicted survival time less than 3 months were excluded. The predicted survival time of patients with spinal metastases was evaluated according to the revised Tokuhashi scoring system^12^.

### Quality of Life Assessment

The European Organization for Research and Treatment of Cancer Quality of Life Questionnaire for Patients with Bone Metastases (EORTC QLQ-BM22) is used to assess disease symptoms related to bone metastases^13^, ^14^. As a PRO measure for patients with bone metastases, it has been translated to Chinese version and has been validated in Chinese population for measuring QoL^15^. The questionnaire comprises 22 items and 4 scales for the measurement of pain in different parts of the body (painful sites), pain characteristics (persistent pain, recurrent pain), functional impairment (occurrence of pain when performing different activities, interference with everyday activities), and psychosocial aspects (family, worries, hope). All items were graded from 1 point (not at all) to 4 point (very much). A higher grade indicates greater distress in symptom scales while a higher grade in functional scale indicates greater functional outcome. Each grade was converted to a score ranging from 0 to 100.

### Surgical Technique

General anesthesia was performed in each patient. Then the patient was placed in the prone position, a midline incision was performed extending over the two lower and two upper vertebral over the lesion level. Exposure of posterior portions of the vertebra was made. Intraoperative C-arm visualization was used to detect the accurate vertebral level.

Initially, transpedicular screws were placed into the two vertebrae upper and lower the damaged vertebra. A rongeur was used to remove the posterior spinous processes. Subsequently, laminectomy was performed by laminectomy rongeur. A temporary rod can be placed on one side after the removal of the posterior complex, which provides temporary stabilization against the instability caused by posterior complex decompression and protect nerve tissues from damage caused by unintended vibrations during manipulation.

Bilateral laminectomy, facetectomy, and wide foraminotomy were performed on the lower and upper levels of the damaged vertebra. The intervertebral disc spaces of the damaged vertebra were emptied and 360 degree debridement was performed either directly via the tissue mass or via the spaces between the nerve roots with a transpedicular approach.

After complete decompression of the neural structures and removal of the tumor, vertebral cement augmentation was performed to reconstruct the anterior column of the spine. Finally, the screws above and below the involved vertebral segment and the rod were fixed, and the system was stabilized posteriorly.

### Follow-up and Data Collection

The Chinese-language version of the EORTC QLQ-BM22 questionnaire was administered at baseline (before surgery) and at one, three, six, and twelve months postoperativelly. The patients received and returned the questions by a questionnaire software or paper version. Moreover, the Karnofsky performance score was used to present the general condition of patients. The visual analog scale (VAS) was used to evaluate pain, and Frankel grade was utilized to assess neurological function.

### Statistical Analyses

Statistical analyses were carried out by using SPSS software, version 22.0 (Armonk, NY, USA). Normally distributed data were recorded using the means ± standard deviation (SD), while nonnormally distributed data were presented as the medians and range. Paired t-tests were used to compare the scores of each scale of the EORTC QLQ-BM22 at one, three, six, and twelve months with the baseline score. Preoperative and postoperative VAS scores were compared by using paired t-test. Kaplan-Meier curves were obtained, the date of surgery was the starting date, and death or the last known contact date was the censor date. A *P* value < 0.05 was considered significant, and a power analysis was conducted.

## Results

### Preoperative Demographic Data

There were 27 male and 12 female with an average age at the time of surgery of 60 years (SD, 14; range, 28 to 92 years). The most common origin of the primary tumor was the liver in 20.5%, followed by the lung, breast and kidney in 15.4% of cases each (Table 1). Two cases had MESCC from an unknown primary site of origin. The median time from the diagnosis of the primary cancer to spinal metastasis was 32 months (SD, 77; range, 0 to 480 months). Twelve patients had multimetastatic disease to visceral organs at the time of surgery. The average Karnofsky performance score was 47, ranging from 20 to 90.

### Surgical Data

All patients underwent planned surgeries and there was no intraoperative death. The mean operative duration was 243 minutes (range, 130 to 450 minutes). The mean intraoperative blood loss was 1417 mL (range, 200 to 3500 mL). No notable intraoperative complications were observed. Three patients experienced cerebrospinal fluid leakage postoperatively, and they were all successfully managed by lying flat without a pillow. One patient experienced rod breakage at 10 months after surgery who received a revison surgery.

### Postoperative Outcomes

**Figure 1:**
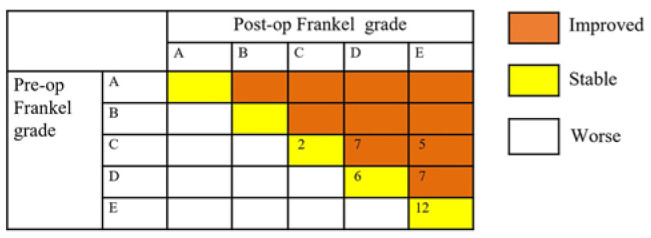
Changes in pre- and postoperative Frankel grades.

All patients showed either an improvement or a similar pain level after surgery (Table 2). The VAS score decreased from 7.10 ± 2.22 preoperatively to 3.10 ± 2.15 one month postoperatively (*P*<0.05). As shown in Figure 1, The Frankel grades decreased significantly after operation. Among 39 patients, preoperatively, 3 were evaluated as Frankel grade B, 5 as Frankel grade C, 11 as Frankel grade D, and 2 as Frankel grade E. Postoperatively, 19 patients (48.7%, 19/39) showed neurological function improvement. Among the 19 patients, 7 cases improved from Frankel grade C to D, 5 cases from grade C to E, and 7 cases from grade D to E. Another 20 patients still have the same Frankel grade postoperatively, however, most of them improved clinically.

The QoL improvement of the patients was also evident after treatment. The EORTC QLQ-BM22 scores of the patients before treatment and 1-month (39 cases), 3-months (39 cases), 6-months (32 cases), and 12-months (23 cases) after treatment are shown in Table 3. Paired-samples T-test examination of the postoperative scores showed a significant improvement in terms of pain location, pain severity and performance status (*P*<0.01). The painful sites scores were 14.5 (SD, 12.4), 13.5 (SD, 12.9), 11.9 (SD, 13.1) and 11.0 (SD, 10.8) at the 1-, 3-, 6-, and 12-months after surgery, respectively. Additionally, painful characteristics were more pronounced in this group of patients, which were 26.5, 25.6, 18.1 and 16.4 at 1-, 3-, 6-, and 12-months after treatment, respectively, compared to 53.0 preoperatively. With regard to functional interference, there was significant improvement one month postoperatively compared with the preoperative score (63.6 vs. 34.5, *P*<0.01). As shown in Table 3, there were no significant changes in social or psychological functioning.

### Survival

**Figure 2:**
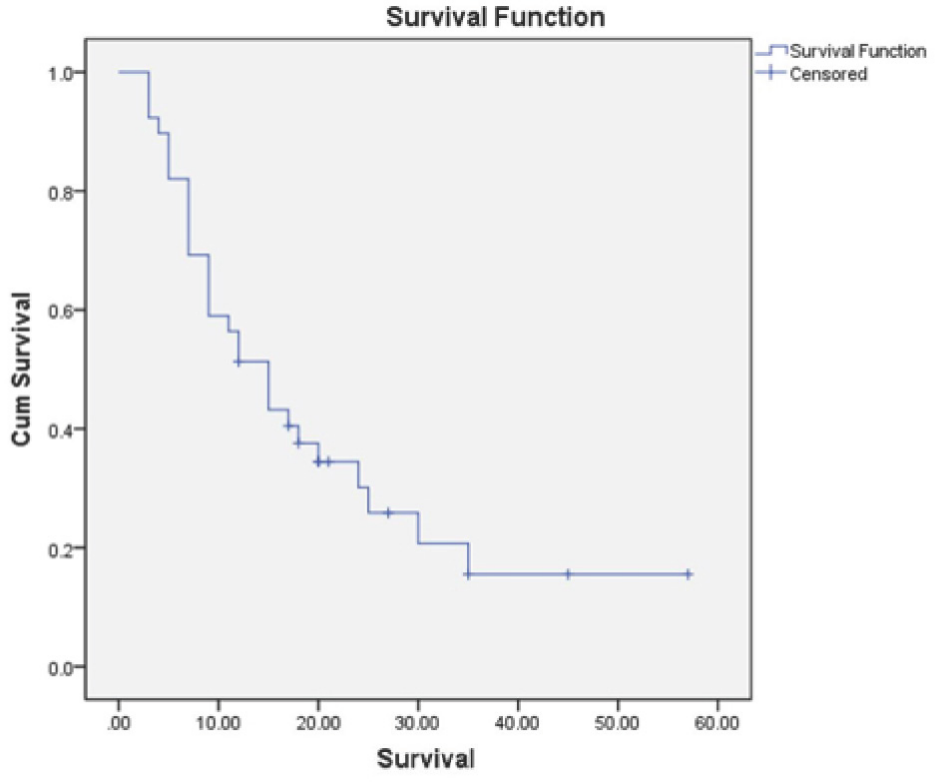
Kaplan-Meier overall survival curve of 39 patients.

The mean follow-up was 15.9 months (SD, 12 months; range, 3 to 57 months). All the patients were alive at 3 months; at 3 to 6 months 7 patients were dead, and at 6 to 12 months, another 9 patients died of disease. During follow-up, 29 patients died. The K-M survival estimate at 1 year postoperatively was 51.3%, and that at 2 years was 30.2% (Figure 2).

## Discussion

The incidence of metastatic spine tumors has increased considerably as a result of the technological development of early diagnosis methods^16^. The expected survival time of those with malignant tumors also been prolonged in recent decades. Decompression and stabilization are indicated in patients with spinal metastases or nonpathological fractures who have high-grade epidural compression with or without neurological deficits and three-column instability. Since most treatment for bone metastasis are palliative in nature, health-related QoL (HRQoL) is theoretically a more meaningful end point together with symptom control, when compared with traditional end points such as survival times and local control 17. HRQoL focused on the patient's feelings and experience after treatment. HRQoL issues are of critical importance for patients, when making decisions in the treatment of bone metastases. To date, there are few prospective studies on surgical outcomes and changes in QoL after surgery in patients with spinal metastases 5, 18, 19. Therefore, we conducted this prospective study to investigate the change in HRQoL in a consecutive series of patients who underwent palliative surgery for vertebral metastases.

Compared with radiotherapy, external irradiation, or minimally invasive procedures such as vertebroplasty or kyphoplasty, open surgery has the conceptual benefit of providing direct spinal cord decompression, reduction of local tumor burden, and the opportunity for mechanical stabilization of the diseased spine. In addition, surgical decompression provides cytoreduction and a margin around neural elements, that is, separation surgery, allowing subsequent adjuvant therapy, which is associated with improved local tumor control^20^-^23^.

The various surgical approaches used to treat spinal metastasis include the following: anterior transcavitary and posterior, posterolateral (transpedicular, costotransversectomy, lateral extracavitary), and a combined approach. Because tumor location, the type of reconstruction needed, patient comorbidities, the extent of primary tumor, and the surgeon's preference have a great impact on the surgical approach, it is essential that the best surgical approach from the patient's perspective be carefully considered before surgery. In the past, many published retrospective studies have shown that 360 degree decompression with a single PTA provides circumferential decompression and stabilization simultaneously, which is associated with less morbidity in systemically compromised patients^10^, ^24^. The present study, based on 39 consecutive patients enrolled with spinal metastases, shows that 360 degree decompression with a single PTA results in significant improvements in pain, neurological function and HRQoL.

Patient-reported HRQoL outcome measures convey information that can be critical in clinical decision making. Among other HRQoL-measuring instruments, the EORTC QLQ-BM22 can effectively detect changes in QoL in diverse bone metastasis populations. This study shows that decompression with a single PTA improves pain, neurologic and functional status, and that EORTC QLQ-BM22 can measure health utilities and HRQoL for symptomatic patients. Compared with preoperative scores, postoperative EORTC QLQ-BM22 scores for painful sites, pain characteristics, and functional interference scale were significantly lower at the 1-month and 3-, 6-, and 12-month follow-ups. As investigators mention in several other studies, spinal surgery for MESCC is associated with rapid, substantial, and sustained pain relief^5^, ^18^. Healthcare professionals will be able to reliably assess their patients' QoL, help patients choose a treatment and assess the cost-effectiveness of the treatments. QoL outcomes in patients with bone matastasis will be compared across clinical trials through the utilization of a consistent and valid questionnaire.

In a previous study, researchers reported that the Eastern Cooperative Oncology Group (ECOG), the Short Form 36 Health Survey (SF-36), the VAS scale, and the assessment of ambulatory status and motor function effectively evaluated the factors that influence the health status of patients with spinal neoplastic disease. In a retrospective study, Chen et al. 25 reported that the EORTC QLQ-BM22 questionnaire showed improved QoL outcomes in patients with spinal metastasis after microinvasive surgery combined with intraoperative radiotherapy. Our results support the fact that surgery improves function and QoL outcomes in patients with metastatic thoracic and lumbar tumors.

In this study, the psychosocial aspects in the EORTC QLQ-BM22 did not reach statistical significance after surgery; which is predominantly due to the small sample size. In addition, patients had different concerns. For example, some patients worried less about a potential loss of mobility, and more about economic factors. Some patients were more worried about becoming dependent on others. Moreover, patients with MESCC represent a frail population with few reserves to deal with additional physical or emotional stress. In future studies, more cases should be included so that each psychosocial items for each individual question can be analyzed to identify areas in which differences are particularly apparent.

This study has several limitations. Although this is a prospective single center study, it is limited by its small sample size and lack of controls. Second, there is no stratification by pathological type due to the small sample of patients. Prospective clinical studies are necessary to further investigate the long-term treatment outcomes of surgery for spinal metastases.

Nonetheless, the present feasibility study found that the application of the posterolateral transpedicular approach for decompression and fusion in patients with spinal metastases can also benefit patients with MESCC in that it achieves prompt and sustained pain relief, reduceds neurolofic deficits and improves functional outcomes, health utilities and HRQoL. Given the overall low incidence, clinical heterogeneity, and rapid development of more efficient medical therapies for the treatment of MESCC, we support current efforts to collect prospective longitudinal data on spine oncology patients.

## Author contributions

JFN: Designing the study, Collecting and analyzing the data, Preparing the manuscript.

ZQZ: Designing the study, Collecting and analyzing the data, Preparing the manuscript.

JCW: Collecting and analyzing the data, Preparing the manuscript.

TQY: Designing the study, Analyzing the data, Preparing the manuscript, Supervision.

WG: Collecting the data, Preparing the manuscript.

RLY: Collecting the data, Preparing the manuscript.

XDT: Collecting the data, Preparing the manuscript.

## Figures and Tables

**Table 1:** Demographic Data of the Patients

No.	Sex	Age	Tumor level	Tumor Pathology	Interval time	Visceral metastasis	KPS
1	F	65	L3	Lung	0		60
2	M	47	L5	Liver cancer	0		60
3	M	66	L3	Unknown	36		40
4	M	49	L3	Lung	0		50
5	F	66	L4	Renal carcinoma	24		50
6	M	69	L3	Renal carcinoma	36		40
7	M	82	T4	Prostate carcinoma	2		40
8	M	78	T11	Liver cancer	23	Liver	50
9	F	46	T5, T6	Breast carcinoma	72		70
10	F	63	L3	Thyroid carcinoma	84	Lung	50
11	F	53	L1	Breast carcinoma	56		60
12	M	61	T5, T6,	Urothelial carcinoma	24	Liver	30
13	F	68	T2	Breast carcinoma	0		30
14	F	72	T8, T9	Breast carcinoma	18		50
15	M	69	T3	Renal carcinoma	36		40
16	M	64	T6	Rectal	0		30
17	M	60	T2	Rectal	22	Liver	40
18	F	43	T4	Breast carcinoma	16		30
19	M	60	L4	Lung	12	Liver	50
20	M	60	L3	Rectal	64	Lung	90
21	M	59	T9	Renal carcinoma	84	Lung\brain	80
22	M	70	T12	Liver cancer	6		50
23	F	72	L4	Leiomyosarcoma of uterus	48	Lung	40
24	M	36	L1	Liver cancer	3	Liver	80
25	M	67	T12	Liver cancer	12		50
26	M	28	T4、T5	Lung	0		50
27	M	56	L2	Thyroid carcinoma	0		50
28	M	58	T3	Renal carcinoma	24		70
29	F	49	L1	Cervical cancer	35		40
30	M	49	T12	Liver cancer	9		50
31	M	70	L2	Unknown	0		40
32	F	58	T9, T11	Myeloma	0		20
33	M	92	T4	Renal carcinoma	7		40
34	M	33	T5, T12	Liver cancer	0		30
35	M	56	T6	Rectal	6		40
36	F	71	T3	Breast carcinoma	480		50
37	M	82	L3	Lung	0	Liver	30
38	M	57	T11	Lung	0	Mediastinum	50
39	M	39	T6	Liver cancer	21	Liver	30

M: Male; F: Female

**Table 2:** The surgical outcome of all patients

No.	Pre-op VAS	Post-op VAS	Pre-op Frankel	Post-op Frankel	Followup Time (month)	End
1	6	2	E	E	7	DOD
2	7	3	E	E	35	DOD
3	7	3	C	D	57	AWD
4	8	8	D	D	7	DOD
5	7	4	D	D	18	DOD
6	8	1	C	D	7	DOD
7	1	0	C	D	35	AWD
8	7	1	D	E	17	DOD
9	7	3	E	E	15	DOD
10	7	6	D	D	25	DOD
11	7	5	D	E	45	AWD
12	7	5	D	E	11	DOD
13	8	4	C	D	12	NED
14	8	3	E	E	15	DOD
15	8	1	C	E	5	DOD
16	8	2	C	E	27	AWD
17	10	3	D	D	5	DOD
18	5	2	D	E	30	DOD
19	7	2	E	E	9	DOD
20	3	1	E	E	24	DOD
21	2	1	E	E	20	DOD
22	9	2	D	E	12	DOD
23	10	7	D	D	15	DOD
24	7	5	E	E	4	DOD
25	7	3	D	E	3	DOD
26	9	6	E	E	9	DOD
27	7	0	C	E	18	AWD
28	5	2	E	E	5	DOD
29	10	6	C	D	12	DOD
30	7	3	E	E	3	DOD
31	9	6	C	C	3	DOD
32	7	4	C	D	17	AWD
33	1	0	C	D	20	AWD
34	8	2	C	E	9	DOD
35	8	7	C	C	7	DOD
36	10	1	D	E	20	AWD
37	10	0	D	D	21	AWD
38	7	4	E	E	9	DOD
39	8	3	C	E	7	DOD

DOD: Died of disease; AWD: Alive with disease; NED: No evidence of disease

**Table 3:** Patients' health-related quality of life evaluation by EORTC QLQ-BM22

Scale	Before surgery	Follow-up at 1 months (N=39)	Follow-up at 3 months (N=39)	Follow-up at 6 months (N=32)	Follow-up at 12 months (N=23)
Painful sites	33.8±20.3	14.5±12.4*	13.5±12.9*	11.9±13.1*	11.0±10.8*
Painful characteristics	53.0±29.2	26.5±23.6*	25.6±25.8*	18.1±18.5*	16.4±16.0*
Functional interference	34.5±28.3	63.6±24.1*	67.6±21.7*	71.2±20.1*	69.9±17.9*
Psychosocial aspects	56.4±21.8	53.3±16.6	54.0±16.3	49.3±17.3	47.6±16.4

* *P*<0.05 compared with the scores before surgery

## Abbreviations

PTA: Posterior transpedicular approach
QoL: Quality of Life
PRO: Patient reported outcome
MESCC: Metastatic epidural spinal cord compression
EORTC QLQ-BM22: The European Organization for Research and Treatment of Cancer Quality of Life Questionnaire for Patients with Bone Metastases
VAS: Visual analog scale
SD: Standard deviation
HRQoL: Health-related QoL
ECOG: Eastern Cooperative Oncology Group
SF-36: Short Form 36 Health Survey
